# Prevalence and predictors of Axis I disorders in a large sample of treatment-seeking victims of sexual abuse and incest

**DOI:** 10.3402/ejpt.v7.30686

**Published:** 2016-04-08

**Authors:** Eoin McElroy, Mark Shevlin, Ask Elklit, Philip Hyland, Siobhan Murphy, Jamie Murphy

**Affiliations:** 1 School of Psychology and Psychology Research Institute, Ulster University, Londonderry, UK; 2 National Center of Psychotraumatology, Institute of Psychology, University of Southern Denmark, Odense, Denmark; 3 Department of Psychology, National College of Ireland, Dublin, Ireland

**Keywords:** Childhood sexual abuse, Axis I disorders, help-seeking sample, sexual trauma, incest, multivariate analysis, multiple perpetrators, adult psychopathology

## Abstract

**Background:**

Childhood sexual abuse (CSA) is a common occurrence and a robust, yet non-specific, predictor of adult psychopathology. While many demographic and abuse factors have been shown to impact this relationship, their common and specific effects remain poorly understood.

**Objective:**

This study sought to assess the prevalence of Axis I disorders in a large sample of help-seeking victims of sexual trauma, and to examine the common and specific effects of demographic and abuse characteristics across these different diagnoses.

**Method:**

The participants were attendees at four treatment centres in Denmark that provide psychological therapy for victims of CSA (*N*=434). Axis I disorders were assessed using the Millon Clinical Multiaxial Inventory-III (MCMI-III). Multivariate logistic regression analysis was used to examine the associations between CSA characteristics (age of onset, duration, number of abusers, number of abusive acts) and 10 adult clinical syndromes.

**Results:**

There was significant variation in the prevalence of disorders and the abuse characteristics were differentially associated with the outcome variables. Having experienced sexual abuse from more than one perpetrator was the strongest predictor of psychopathology.

**Conclusions:**

The relationship between CSA and adult psychopathology is complex. Abuse characteristics have both unique and shared effects across different diagnoses.

**Highlights of the article:**

- The prevalence of Axis I disorders were assessed in a large sample of sexual abuse and incest survivors.
- The impact of demographic and abuse characteristics were also examined.
- There was significant variation in the prevalence of disorders.
- Abuse characteristics were differentially associated with the disorders.
- Abuse from multiple perpetrators was the strongest overall predictor of psychopathology.

Childhood sexual abuse (CSA) is not a rare occurrence. Lalor and McElvaney (2010) summarised the findings from many surveys of child sexual abuse across European countries and reported prevalence rates of 13.9% (Sweden), 15.8% (Denmark), and 19% (Spain) for females and 6.7% (Denmark), 15.2% (Sweden), and 15.5% (Spain) for males. Further, a recent meta-analysis estimated the global prevalence of CSA to be 11.8% based on 217 studies (Stoltenborgh, Van Ijzendoorn, Euser, & Bakermans-Kranenberg, 2011). The variability in estimates of CSA is due to inconsistent definitions of what constitutes abuse, different sampling methods, and the types of data that are used (e.g., self-report or secondary data).

CSA is a robust predictor of adult psychopathology (Anda et al., 2006; Hillberg, Hamilton-Giachritsis, & Dixon, 2011; Kendall-Tackett, Williams, & Finkelhor, 1993; Maniglio, 2009). The nature of this relationship, however, remains remarkably non-specific. Indeed, CSA has been linked with the development of a wide variety of adult psychiatric disorders, including mood and anxiety disorders, substance dependence, posttraumatic stress disorder, and psychosis (Bebbington et al., 2011; Cutajar et al., 2010; Fergusson, Boden, & Horwood, 2008; Fergusson, Horwood, & Lynskey, 1996; Fergusson, McLeod, & Horwood, 2013; Kessler, Davis, & Kendler, 1997; Molnar, Buka, & Kessler, 2001; Read, Agar, Argyle, & Aderhold, 2003; Varese et al., 2012). The relationship between CSA and adult psychopathology is further complicated by the influence of a variety of demographic factors (e.g., gender and age when abused) and abuse characteristics (e.g., duration, number of abusive acts, number of abusers, concurrent physical abuse and neglect). The common and /or shared effects of such factors are yet to be firmly established.

Indeed, a large body of research has examined the influence of gender on the relationship between CSA and adult psychopathology, yet the findings have been contradictory. Some studies have suggested that the effects of CSA are more serious for men (Garnefski & Diekstra, 1997; Gold, Lucenko, Elhai, Swingle, & Sellers, 1999) while others have suggested women experience more negative outcomes (Putnam, 2003). Moreover, a number of reviews and meta-analyses have found no effect of gender (Chen et al., 2010; Fergusson et al., 2013; Hillberg et al., 2011). The equivocal role that gender plays could be due to the influence of other variables. For example, in their analysis of the British Adult Psychiatric Morbidity Survey, Jonas et al. (2011) found that gender moderated the impact of CSA on a variety of common mental health disorders only in cases where the most severe form of CSA (penetrative sex) had occurred. In this case, odds ratios (ORs) for a variety of common mental health disorders were significantly higher for females (Jonas et al., 2011). This finding suggests that gender may play a role, however, only when combined with certain abuse characteristics. It is important to note, however, that abuse characteristics vary depending on gender. For example, Maikovich-Fong and Jaffee (2010) found that girls were more likely to experience penetrative sexual abuse. As such, it is possible that gender effects are attributable to the increased likelihood of penetrative sex associated with being female. Further research is required to understand the interplay between these factors.

The age at which a child is first abused is another demographic factor that has been proposed to have an impact on adult psychopathology. Again, studies have produced mixed findings. A common assumption is that abuse at an earlier age is associated with greater psychological dysfunction in adulthood (Cutajar et al., 2010). In actuality, the opposite pattern has been observed in research. Indeed, a number of studies suggest that the negative outcomes associated with CSA are greatest when abuse occurs in adolescence (Cutajar et al., 2010; Finkelhor, Ormrod, & Turner, 2007; Thornberry, Ireland, & Smith, 2001). For example, Cutajar et al. (2010) examined the psychopathology of a large sample of CSA survivors (*N=*2,759) over a 43-year period. Their data came from forensic medical records and cases were compared with a general population control group. They found that older age of abuse was associated with a greater risk for adult psychopathology. Other studies have suggested that psychiatric outcomes in adulthood may vary depending on the developmental period during which CSA occurs. For instance, Schoedl et al. (2010) examined the impact of CSA on adult depression and posttraumatic stress disorder (PTSD) sample of psychiatric inpatients (*N*=60). They found that those who reported CSA before age 12 were more likely to meet the diagnostic criteria for major depression, while those abused after 12 years were 10 times more likely to develop PTSD. It must be noted, however, that not all studies support the effect of age; Paolucci, Genuis, and Violato (2001) conducted a meta-analysis of 37 studies (*N*=25,367), yet found no aggregated effect of age on the relationship between CSA and adult psychopathology. As such, further research is required to improve our understanding of the impact of CSA at different developmental periods.

While the effects of demographic factors remain debated, there is more consistent evidence that abuse characteristics influence the relationship between CSA and adult psychopathology. In general, there is evidence of a dose–response relationship, with more severe abuse appearing to have a more deleterious impact on adult mental health (Hovens et al., 2010; Kendall-Tackett et al., 1993; Sugaya et al., 2012). What constitutes severe abuse, however, is open to interpretation and many abuse characteristics must be considered. Research has examined the impact of different types of CSA on adult mental health. Previous studies have generally taken an ordered categorical approach, with cases assigned to a category based on the most severe type of abuse suffered. While there is significant heterogeneity in the CSA categories utilised, they tend to fall into one of four groupings; non-contact (suggestive talking, showing pornography, exposing), non-genital contact (e.g., kissing, fondling), genital contact (e.g., touching, masturbation), and CSA involving penetrative intercourse. This approach has delivered consistent findings, with non-genital CSA demonstrating a statistically non-significant or minor influence on later psychopathology, and CSA involving intercourse leading to significantly higher levels of psychological distress (Bulik, Prescott, & Kendler, 2001; Fergusson et al., 2008; Kendler et al., 2000; Molnar et al., 2001; Nelson et al., 2002). To our knowledge, no studies have examined the impact of multiple types of CSA on adult mental health. Given that it would be likely for other forms of CSA to accompany penetrative sex, the unique impact of penetrative CSA may have been overstated in previous research. As such, research examining the impact of multiple types of CSA is warranted.

A number of studies have demonstrated that the negative outcomes associated with CSA are more severe when children are abused by multiple perpetrators (Cutajar et al., 2010; Liu et al., 2012; Steel, Sanna, Hammond, Whipple, & Cross, 2004). For example, Cutajar et al. (2010) found that children who had suffered abuse at the hands of more than one person were almost twice as likely to receive a diagnosis of an Axis I disorder or engage with adult mental health services in adulthood. This effect was found while controlling for a variety of other abuse characteristics including penetration versus non-penetration, age at abuse, and gender (Cutajar et al., 2010). Similarly, Steel et al. (2004) observed a direct association between the number of perpetrators and adult psychological distress in a sample of psychiatric outpatients and inpatients (*N*=285). These findings provide convincing evidence that abuse by multiple offenders has a more negative impact on adult mental health than abuse by a single perpetrator.

Certain abuse characteristics appear to have a more ambiguous impact on adult mental health. A plethora of research has examined the impact of duration, with the assumption that sustained CSA would be more detrimental than an isolated incident. Surprisingly, the findings have been unclear. A number of studies have found evidence that CSA duration is associated with higher levels of adult psychopathology (Hovens et al., 2010; Steel et al., 2004). For example, Steel et al. (2004) tested the association between abuse characteristics and psychological distress through a variety of mediating factors such as seeking social support and coping strategies (*N*=285). They found that duration was one of only two abuse characteristics (the other being number of offenders) to have a direct association with psychological distress in adulthood. Other studies have found no effect of abuse duration (Friedrich, Urquiza, & Beilke, 1986; Tyler, 2002). Friedrich et al. (1986) examined behavioural problems in a sample of sexually abused children (*N*=85). While they found that the duration of abuse correlated with the severity of behavioural problems, when entered into a multiple regression analysis, duration did not explain any unique variance. This suggests that the impact of duration may be explained by other related abuse characteristics, such as frequency and severity, with abuse lasting for longer periods more likely to be frequent and severe. These conflicting findings indicate that further research is required to determine whether the duration of abuse makes a unique contribution to the relationship between CSA and adult psychopathology.

Research in this area has often faced criticism with regards to the assessment of CSA. In the majority of general population and clinical studies, CSA is self-reported based on retrospective recall. Such methods may be subject to recall bias (Fergusson et al., 2013; Widom & Morris, 1997). In the case of childhood trauma, the literature suggests that type II errors (i.e., failing to report an incidence of CSA) are more likely to occur than type I errors (i.e., reporting CSA when it did not occur) (Hardt & Rutter, 2004; Widom & Morris, 1997). This bias could be due to a number of reasons, for example, forgetting, re-evaluating the actions of others based on current knowledge, and embarrassment or un/conscious denial (Widom & Morris, 1997). In order to eliminate or at least minimise such bias, some researchers have suggested using samples of people with official histories of CSA, such as those referred to sexual trauma clinics (Widom & Morris, 1997). Data acquired from such sources is likely to be more reliable and objective.

A disadvantage of using the above method relates to sample size, as samples of confirmed victims tend to be relatively small. Indeed, Kendall-Tackett et al. (1993) conducted a meta-analysis of 45 studies of CSA survivors, with average sample sizes of between 25 and 50. A number of problems are associated with using small samples, such as lower statistical power and a lack of generalisability (Button et al., 2013). Moreover, studies employing small samples may lead to exaggerated effects due to publication bias (Klonsky & Moyer, 2008). Studies have also been criticised for neglecting other forms of childhood trauma (e.g., physical abuse and neglect) that frequently occur along with CSA (Felitti et al., 1998; Green et al., 2010; Kessler et al., 2010; Sugaya et al., 2012). A growing body of research suggests that multiple forms of childhood abuse, also known as poly-victimisation, may increase the risk for adult psychopathology (Finkelhor et al., 2007; Turner, Finkelhor, & Ormrod, 2010; Shevlin, McElroy, & Murphy, 2015). For example, in their study of a nationally representative sample of children, Finkelhor et al. (2007) found that poly-victimisation was highly predictive of trauma symptoms and that it also greatly reduced or eliminated the association between individual forms of abuse (e.g., CSA) and trauma symptoms. As such, any research looking at the impact of CSA on adult mental health should take other types of childhood trauma into account, as focussing on only one type of trauma may exaggerate any effects that are found.

This study sought to address the above limitations by assessing the prevalence of Axis I disorders in a large sample of help-seeking victims of sexual trauma, while controlling for other forms of childhood trauma. In addition, demographic (age and gender) and trauma-related variables (age of first abuse, duration of abuse, number of abusive acts, one or more perpetrators) were used to determine if these risk factors had common (or specific) effects across different diagnoses.

## Methods

### Participants

The participants in this study were all male and female consecutive attendees (*N*=434) at four treatment centres in Denmark that provide psychological treatment for victims of CSA and incest. The centres are supported by the Ministry of Social Affairs. Exclusion criteria were (1) presenting under the influence of alcohol or drugs, (2) a diagnosis of a psychotic disorder, (3) self-harming behaviour, (4) engagement in treatment elsewhere, and (5) diagnosis of a personality disorder. Clients who met one or more of the exclusion criteria were referred either to specialised institutions or voluntary help groups. Most of participants were women (85.5%), the mean age was 36.62 years (SD*=*10.74), and most (50.9%) were either married or cohabiting. The average length of education was 13.30 years (SD=3.50). The majority of participants were abused by a family member (83.3%). Prior approval for the present study was obtained from the relevant university ethical boards in Denmark.

### Procedure

When clients initially attended the treatment centre, they were informed that they would be asked to fill out a number of questionnaires during their second session, based on which the therapy would be planned. The therapist shared the findings with the client during the following session. The present study is based on information from the questionnaires. All of the survivors received individual therapy.

### Measures

Axis I diagnoses were assigned based on the Millon Clinical Multiaxial Inventory-III (MCMI-III; Millon, Millon, Davis, & Grossman, 2009). The MCMI-III is a self-reported measure of psychopathology. It consists of 175 true–false questions that measure 14 personality disorder scales (Axis II disorders) and 10 clinical syndromes (Axis I disorders) that correspond to DSM-IV nosology (MCMI-III; Millon et al., 2009). This study used only scores from the clinical syndrome scales (Anxiety Disorder, Somatoform, Bipolar: Manic Disorder, Dysthymic Disorder, Alcohol Dependence, Drug Dependence, Posttraumatic Stress Disorder, Thought Disorder, Major Depression, Delusional Disorder). Prevalence of pathology on the MCMI-III was estimated using base rate (BR) scores that range from 0 to 115. A cut-off score of 75 or more for each of the Axis I clinical syndrome scales was used to be indicative of a probable Axis I diagnosis. Profiles that did not fulfil the MCMI-III validity criteria were excluded. Psychometric research indicates that it is a reliable and valid diagnostic tool in clinical and general population samples and process of translating and validating the Danish version of the MCMI-III has been well documented (Craig, 2013; Ravndal & Vaglum, 2009; Rossi, Van den Brande, Tobac, Sloore, & Hauben, 2003; Simonsen & Elklit, 2008).

Participants were also asked to complete a questionnaire that assessed demographic variables (age, gender, marital status, and education) and sexual trauma variables. The 18 types of abuse were classified into three types: non-contact (spoken to about sexual matters, questioned about sexuality, teased about sexual development, had to listen to other's sexual experiences, proposals or threats about taking part in sexual acts, watch someone present their genitals, watch adult intercourse or pornographic material, present own genitals to someone else); non-penetrative contact (kissed or fondled in a sexual way, touched in a sexual way (non-genital), genitals were touched in a sexual way, had to touch or fondle the genitals of someone else, had to masturbate while someone was watching, reciprocal masturbation); and penetrative contact (attempted intercourse, oral intercourse, anal intercourse, genital intercourse). These questions were answered “Yes” (1) or “No” (0), and the responses were summed to represent the total number of different abusive acts.

A list of perpetrators was provided and the participants were asked to indicate who had been involved in the abuse. A binary variable was computed that indicated if the participant had been abused by one (0) or more than one (1) perpetrator. The participants were also asked what age they were when the abuse started, and how long the abuse lasted (years). The participants were also asked if they have been victims of childhood physical abuse or childhood neglect; both items were scored “Yes” (1) or “No” (0).

### Analysis

Multivariate logistic regression analysis was carried out using Mplus 6.1 (Muthen & Muthen, 2010). Multivariate regression analysis was utilised in this study as it allows several dependent variables to be jointly regressed on predictor variables. This reduces the likelihood of a Type I error compared with conducting separate analysis for each dependent variable. The predictor variables in this model were age, gender, age when the abuse started, duration of abuse (years), the number of abusive acts they experienced, whether there were one or more abusers, and childhood physical abuse and neglect. Robust maximum likelihood (MLR) estimation was employed (Satorra & Bentler, 1994) and the estimates were presented as ORs.

## Results

The average age at which the abuse started was 6.57 years (SD=4.70) and this did not differ significantly for males and females, *t*(372)=1.70, *p*>0.05. The abuse lasted for an average of 6.88 years (SD=6.39) and this did not differ significantly for males and females, *t*(305)=−0.52, *p*>0.05. Only 4.8% of the participants reported that they had experienced a single abusive act (M=7.16, SD=4.08), and this did not differ significantly for males and females, *t*(400)=0.17, *p*>0.05 (SD=4.15). Almost a quarter (24.9%) of the sample reported being victimised by more than one person, with more females (27.5%) than males (10.9%) reporting this, *χ* ^2^ (1)= 7.93, *p*<0.05. Those who were abused by multiple perpetrators suffered a significantly greater number of abusive acts than those who were abused by just one person, *t*(468)=−2.83, *p*<0.001. Rates of childhood neglect (61.8%) were higher than childhood physical abuse (31.7%), and there were no significant gender differences.

Table 1 shows the prevalence of clinically significant syndromes for each of the MCMI clinical scales. There was significant variation in the prevalence of disorders. There were high rates of Anxiety Disorder (76.3%), Dysthymic Disorder (39.9%), and Major Depression (37.1%). Rates of Alcohol (5.5%) and Drug Dependence (4.1%) were relatively low. The chi-square statistics indicated that males were significantly more likely to meet the criteria for Dysthymic Disorder, Alcohol and Drug Dependence, and Thought Disorder while females had higher rates of Somatoform Disorder and Major Depression. There were no significant sex differences with regards to the diagnosis of anxiety disorder, bipolar disorder, PTSD, or delusional disorder.

**Table 1:** Prevalence of Axis I syndromes for male and female victims of sexual victimisation

	Female (*N*=360)	Male (*N*=74)	Total	*χ* ^2^ (df) *p*
Anxiety disorder	273 (75.8%)	58 (78.4%)	332 (76.3%)	0.22 (1) 0.64
**Somatoform**	**82 (22.8%)**	**8 (10.8%)**	**90 (20.7%)**	**5.34 (1) 0.02**
Bipolar: manic disorder	43 (11.9%)	11 (14.9%)	54 (12.4%)	0.48 (1) 0.48
**Dysthymic disorder**	**125 (34.7%)**	**48 (64.9%)**	**173 (39.9%)**	**23.26 (1) 0.00**
**Alcohol dependence**	**7 (1.9%)**	**17 (23.0%)**	**24 (5.5%)**	**51.95 (1) 0.00**
**Drug dependence**	**9 (2.5%)**	**9 (12.2%)**	**18 (4.1%)**	**14.41 (1) 0.00**
Posttraumatic stress disorder	73 (20.3%)	16 (21.6%)	89 (20.5%)	0.06 (1) 0.79
**Thought disorder**	**54 (15.0%)**	**22 (29.7%)**	**76 (17.5%)**	**9.22 (1) 0.00**
**Major depression**	**141 (39.2%)**	**20 (27.0%)**	**161 (37.1%)**	**3.88 (1) 0.04**
Delusional disorder	33 (9.2%)	4 (5.4%)	37 (8.5%)	1.11 (1) 0.29

*Note:* Statistically significant associations (*p*<0.05) in bold.

The results from the multivariate logistic regression analysis are presented as ORs in Table 2. Childhood neglect, respondent age, and age of first abuse did not explain any unique variance in the MCMI diagnoses. Females who experienced CSA were almost three times more likely to be diagnosed with somatoform disorder compared with males. Moreover, being female was associated with significantly reduced odds of being diagnosed with alcohol dependence, drug dependence and dysthymic disorder (ORs ranging from 0.29 for dysthymic disorder to 0.06 for alcohol dependence). Those who suffered more than one type of abusive act were more likely to be diagnosed with anxiety disorder, somatoform disorder, drug dependence, PTSD, and major depression (ORs ranging from 1.07 for major depression to 1.22 for drug dependence). The co-occurrence of CSA and childhood physical abuse was only associated with an increased risk for alcohol dependence (OR=2.89).Those who were abused by multiple perpetrators were more likely to meet the diagnostic criteria for anxiety disorder, somatoform disorder, dysthymic disorder, PTSD, thought disorder, major depression, and delusional disorder (ORs ranging from 2.35 for thought disorder to 3.34 for major depression). Those who experienced abuse over longer time periods were less likely to be diagnosed with anxiety, somatoform, dysthymic, and thought disorders and major depression (ORs ranging from 0.93 for anxiety disorder to 0.87 for thought disorder).

**Table 2:** Odds ratios from the multivariate logistic regression analysis with clinical syndromes predicted by demographic and abuse risk factors

Predictors	Anxiety	Somatoform	Bipolar	Dysthymic disorder	Alcohol dependence	Drug dependence	PTSD	Thought disorder	Major dependence	Delusional disorder
Gender (female)	0.85 (0.41–1.74)	**2.96 (1.11–7.89)**	1.27 (0.52–3.06)	**0.29 (0.15–0.57)**	**0.06 (0.02–0.19)**	**0.21 (0.05–0.85)**	0.78 (0.38–1.60)	0.47 (0.21–1.07)	1.90 (0.90–4.02)	1.14 (0.37–3.49)
Age	0.99 (0.96–1.01)	1.01 (0.98–1.04)	1.00 (0.97–1.03)	1.00 (0.98–1.02)	1.02 (0.98–1.05)	0.99 (0.93–1.06)	1.01 (0.98–1.04)	0.99 (0.97–1.02)	0.99 (0.97–1.01)	1.01 (0.98–1.05)
Age first abuse	0.98 (0.91–1.05)	0.98 (0.89–1.07)	1.02 (0.94–1.11)	0.95 (0.88–1.02)	0.98 (0.82–1.18)	0.93 (0.80–1.09)	0.98 (0.89–1.07)	0.93 (0.83–1.03)	1.01 (0.93–1.09)	0.95 (0.83–1.10)
Duration of abuse (years)	**0.93 (0.89–0.97)**	**0.91 (0.84–0.98)**	0.94 (0.86–1.02)	**0.91 (0.86–0.97)**	0.97 (0.87–1.08)	0.98 (0.85–1.13)	0.93 (0.87–1.00)	**0.87 (0.78–0.96)**	**0.91 (0.85–0.97)**	0.98 (0.91–1.06)
Number of abuse acts	**1.14 (1.06–1.22)**	**1.08 (1.00–1.17)**	1.03 (0.94–1.13)	1.01 (0.95–1.07)	1.02 (0.92–1.14)	**1.22 (1.08–1.38)**	**1.10 (1.02–1.18)**	1.04 (0.96–1.13)	**1.07 (1.00–1.15)**	1.07 (0.97–1.18)
More than one abuser	**2.84 (1.06–7.64)**	**2.91 (1.31–6.44)**	1.50 (0.53–4.22)	**3.14 (1.47–6.70)**	0.37 (0.02–5.44)	0.66 (0.09–4.90)	**2.37 (1.12–5.01)**	**2.35 (1.01–5.50)**	**3.34 (1.49–7.47)**	**2.73 (1.09–6.84)**
Childhood physical abuse	0.92 (0.52–1.65)	1.64 (0.91–2.96)	0.62 (0.28–1.37)	1.22 (0.72–2.06)	**2.89 (1.01–8.24)**	2.17 (0.65–7.18)	1.41 (0.79–2.49)	1.26 (0.64–2.45)	1.54 (0.90–2.64)	1.76 (0.81–3.84)
Childhood neglect	1.58 (0.96–2.61)	1.11 (0.86–1.43)	0.80 (0.47–1.34)	1.18 (0.81–1.72)	0.90 (0.39–2.07)	0.97 (0.41–2.30)	1.10 (0.88–1.37)	1.19 (0.91–1.55)	1.29 (0.71–2.36)	1.35 (1.02–1.80)

*Note:* Statistically significant associations (*p*<0.05) in bold.

## Discussion

The present study sought to assess the prevalence of Axis I disorders in a large sample of help-seeking victims of sexual trauma. First, it is worth noting the high prevalence of MCMI-III diagnoses in this sample. This adds to the already substantial body of evidence that links CSA with a wide range of adult psychiatric disorders (Bebbington et al., 2011; Cutajar et al., 2010; Fergusson et al., 1996, 2008; Fergusson et al., 2013; Kessler et al., 1997; Molnar et al., 2001; Read et al., 2003; Varese et al., 2012).

This study also aimed to examine whether certain demographic and trauma variables had common or specific effects across different diagnoses. In previous studies, victim gender has had an ambiguous impact on psychopathology (Chen et al., 2010; Fergusson et al., 2013; Garnefski & Diekstra, 1997; Gold et al.,^1999^; Hillberg et al., 2011; Putnam, 2003). In the present study, females were significantly more likely to be diagnosed with somatoform disorder and major depression, whereas males were more likely to be diagnosed with dysthymia, substance dependence, and thought disorder. When entered into the multiple regression analysis, however, the effect of gender was no longer significant for thought disorder or major depression. This suggests that associations between gender and major depression and thought disorder could be a result of common effects of abuse characteristics. The effect of gender on somatoform disorder, substance dependence, and dysthymia remained significant when entered into the regression analysis, suggesting that gender has a specific effect on these conditions. Indeed, this is consistent with epidemiological research which indicates that the prevalence of somatoform disorder is higher for women (Rief, Hessel, & Braehler, 2001; Kroenke & Spitzer, 1998), whereas rates of substance dependence and thought disorder are generally higher for men (Aleman, Kahn, & Selten, 2003; Grant et al., 2004). These unique effects of gender could be explained by a variety of biological (e.g., hormonal differences) and/or cognitive (e.g., different coping styles) differences that exist between the sexes (Hyde, Mezulis, & Abramson, 2008; Matud, 2004). As such, it appears that the gender of CSA victims has both common and specific effects on adult psychopathology.

This study also examined the impact of the victim's age when first abused; however, no statistically significant associations were identified between age of abuse and the MCMI-III diagnoses. This is in line with a meta-analysis conducted by Paolucci et al. (2001). They analysed 37 studies of CSA published between 1981 and 1995 (*N*=25,367), yet found no effect of age of abuse on six psychiatric outcomes (PTSD, depression, suicide, sexual promiscuity, victim-perpetrator cycle, and poor academic performance). As such, it would appear that CSA can have a negative impact on adult psychopathology, regardless of when the abuse commenced.

In the present study, the most significant and consistent predictor of adult psychopathology was the presence of more than one abuser in childhood. Indeed, those who had been abused by more than one person were between two and four times more likely to meet the MCMI criteria for PTSD, major depression, anxiety, somatoform, dysthymic, thought and delusional disorders. As such, these findings suggest that the presence of multiple abusers has a unique effect on adult psychopathology. This supports a large body of past research (Cutajar et al., 2010; Liu et al., 2012; Steel et al., 2004). A possible explanation for this effect is that those who are abused by more than one person experience greater levels of shame and distrust, which may impede attempts to disclose the abuse. This in turn may prevent victims from receiving the social support required to buffer the negative impact of CSA (Steel et al., 2004). This finding lends further credence to the overall “dose–response” relationship, and suggests that the number of perpetrators is an important characteristic to consider when attempting to define the severity of CSA. As such, the number of perpetrators may be an important aspect to consider in assessment of overall CSA severity, both in clinical and research contexts. Furthermore, at a clinical level, interventions aimed at addressing feelings of mistrust and guilt associated with multiple perpetrators may prove effective at reducing CSA-related distress.

This study also suggests that the range of abusive acts may be reflective of overall abuse severity. The odds of being diagnosed with anxiety disorder, somatoform disorder, major depression, and substance dependence were higher for those who had experienced more than one form of abusive act. While the effect sizes were relatively small (ORs ranging from 1.07 for major depression to 1.22 for alcohol dependence), these findings suggest that there may be an additive effect of suffering more than one form of CSA. Previous studies of CSA have taken an ordered categorical approach (i.e., assigning subjects to groups based on the most severe form of CSA experienced) (c.f., Bulik et al., 2001; Fergusson et al., 2008; Kendler et al., 2000; Molnar et al.,^2001^; Nelson et al., 2002). As such, it is possible that the effects of penetrative sex (traditionally seen as the most severe form of CSA) have been overstated slightly in previous studies, given that multiple forms of CSA likely co-occur with intercourse. Therefore, future studies may wish to consider the presence of multiple forms of CSA when attempting to assess the overall severity of abuse.

Research also suggests that CSA is frequently accompanied by other forms of maltreatment in childhood, such as physical abuse (CPA) and neglect (Green et al., 2010; Kessler et al., 2010; Sugaya et al., 2012). Indeed, there were high levels of both CPA and neglect reported in this sample. Interestingly, when entered into the multiple regression analysis, CPA only had a statistically significant association with alcohol dependence. This effect was relatively large, with victims of both CPA and CSA almost three times more likely to meet the diagnostic criteria for alcohol dependence. Moreover, the presence of childhood neglect explained no unique variance in the MCMI diagnoses. These findings do not support previous research, which found that exposure to multiple types of childhood trauma (or “poly-victimisation”) led to increased risk for a variety of adult mental health problems, including anxiety, depression, and psychosis (Finkelhor et al., 2007; Turner et al., 2010; Shevlin et al., 2015). It is worth noting that previous studies of poly-victimisation have focussed on the presence/absence of CPA and CSA, rather than examining the impact of specific abuse characteristics (Finkelhor et al., 2007; Turner et al., 2010; Shevlin et al., 2015). This may account for the differing findings; it is possible that the combined effects of CSA and CPA identified in previous studies were attributable to more severe CSA characteristics (e.g., multiple abusers, penetrative sex) that were not adequately captured in the analyses.

The present study also examined the impact of the duration of CSA on adult psychopathology. Surprisingly, the duration of abuse (in years) was *negatively* associated with the development of anxiety, somatoform disorder, dysthymic disorder, thought disorder, and major depression (ORs ranging from 0.87 for thought disorder to 0.93 for anxiety). It is important to note that research examining the impact of CSA duration has been highly inconsistent. Indeed, some studies have suggested a positive association between duration and psychopathology (Hovens et al., 2010; Steel et al., 2004), whereas others have found no such effect (Friedrich et al., 1986; Tyler, 2002). To our knowledge, this is the first empirical study to identify a negative association between CSA duration and psychopathology, albeit the effects were quite small. Perhaps the most plausible explanation for this anomalous finding is that it was a statistical artefact. Indeed, this model included a comprehensive set of predictors. When the variance associated with the more reliably linked predictors was explained, it is possible that this relationship arose due to spurious associations amongst residuals. This is known as a suppressor effect (MacKinnon, Krull, & Lockwood, 2000).

The present study had a number of strengths. First, participants were help-seeking victims of CSA. Using a sample of verified abuse victims likely helped reduce the level of recall bias (Fergusson et al., 2013; Widom & Morris, 1997). Also, the relatively large sample size overcame the problem of statistical power which has been a common criticism of studies of CSA victims (Button et al., 2013; Kendall-Tackett et al., 1993; Klonsky & Moyer, 2008). However, the findings of this study should be considered in light of the following limitations. First, while a broad range of abuse characteristics were included in the analysis, this list was not exhaustive. For example, frequency of CSA and adult revictimisation were not included. Also, it must be noted that this study was cross-sectional in nature. While this was a sample of help-seeking CSA victims and the psychiatric measures were administered well after the traumatic incidents, it cannot be unequivocally established whether the psychiatric disorders were present prior to the abuse. Furthermore, a number of participants were excluded from the present analysis to ensure they received treatment at the appropriate institution (e.g., those presenting under the influence of drugs/alcohol, with self-harming behaviour or with psychotic or personality disorders). This may impact the generalisability of the findings.

In conclusion, the present study assessed the prevalence of Axis I disorders in a large sample of help-seeking victims of sexual trauma. Overall, the prevalence of MCMI disorders was high in this sample, particularly for anxiety disorder, dysthymic disorder, and depression. This study also examined the effects of various abuse and demographic factors across the diagnoses. The presence of multiple abusers had the largest specific effects. Gender, multiple abusive acts, and the co-occurrence of CPA also had unique effects on adult psychopathology. Interestingly, duration of abuse was negatively associated with a variety of disorders. Overall, the relationship between CSA and adult psychopathology is complex and influenced by a variety of demographic and abuse characteristics. The number of abusers and the number of abusive acts may be important aspects to consider when determining the overall severity of CSA.
